# Multisensory guided associative learning in healthy humans

**DOI:** 10.1371/journal.pone.0213094

**Published:** 2019-03-12

**Authors:** Gabriella Eördegh, Attila Őze, Balázs Bodosi, András Puszta, Ákos Pertich, Anett Rosu, György Godó, Attila Nagy

**Affiliations:** 1 Department of Operative and Esthetic Dentistry, Faculty of Dentistry, University of Szeged, Szeged, Hungary; 2 Department of Physiology, Faculty of Medicine, University of Szeged, Szeged, Hungary; 3 Department of Psychiatry, Faculty of Medicine, University of Szeged, Szeged, Hungary; 4 Csongrád County Health Care Center, Psychiatric Outpatient Care, Hódmezővásárhely, Hungary; University of Ontario Institute of Technology, CANADA

## Abstract

Associative learning is a basic cognitive function by which discrete and often different percepts are linked together. The Rutgers Acquired Equivalence Test investigates a specific kind of associative learning, visually guided equivalence learning. The test consists of an acquisition (pair learning) and a test (rule transfer) phase, which are associated primarily with the function of the basal ganglia and the hippocampi, respectively. Earlier studies described that both fundamentally-involved brain structures in the visual associative learning, the basal ganglia and the hippocampi, receive not only visual but also multisensory information. However, no study has investigated whether there is a priority for multisensory guided equivalence learning compared to unimodal ones. Thus we had no data about the modality-dependence or independence of the equivalence learning. In the present study, we have therefore introduced the auditory- and multisensory (audiovisual)-guided equivalence learning paradigms and investigated the performance of 151 healthy volunteers in the visual as well as in the auditory and multisensory paradigms. Our results indicated that visual, auditory and multisensory guided associative learning is similarly effective in healthy humans, which suggest that the acquisition phase is fairly independent from the modality of the stimuli. On the other hand, in the test phase, where participants were presented with acquisitions that were learned earlier and associations that were until then not seen or heard but predictable, the multisensory stimuli elicited the best performance. The test phase, especially its generalization part, seems to be a harder cognitive task, where the multisensory information processing could improve the performance of the participants.

## Introduction

Associative learning is a basic cognitive function by which discrete and often different percepts will be linked together. It contributes to several cognitive tasks, i.e. classical conditioning [1], latent inhibition [2] and sensory preconditioning [3]. Catherine E. Myers and co-workers developed a learning paradigm (Rutgers Acquired Equivalence Test, also known as the fish-face paradigm) that can be applied to investigate a specific kind of associative learning, which is visually guided equivalence learning [4]. This test can be divided into two main phases. In the acquisition phase, the subjects are asked to associate two different visual stimuli as the computer provides information about the correctness of the responses. After that in the test phase the subjects receive no feedback about the correctness of their choices. In the test phase, beside the stimulus pairs learned earlier (retrieval part), hitherto not encountered but predictable associations (generalization part) are also presented. A substantial advantage of this test is that well-circumscribed brain structures play the main role in different phases of the test. Optimal performance in the acquisition phase appears to depend mainly on the integrity of the basal ganglia, whereas performance in the test phase (both retrieval and generalization) has been linked to the integrity of the hippocampal region [4, 5]. Our research group has a particular interest in the sensorimotor and cognitive functions of the basal ganglia and has studied with this paradigm since 2006, mostly to assess the development of visually guided associative learning [6] and to examine the progress in various conditions, from Alzheimer's disease to migraines [7–9]. It is well known from earlier studies that both brain structures fundamentally involved in visual associative learning, the basal ganglia and the hippocampi, receive not only visual but also multisensory information [10–13]. Multimodal information could be more informative than a unimodal stimulus from the environment [14, 15]. Probably because of the merging of senses, multisensority has a priority in spatial orientation and in recognizing objects and events from the multisensory environment [14–16]. Multisensory integration occurs at different levels of brain functions. It can be observed at the cellular level [17–20] in several brain regions such as the superior colliculus [21], basal ganglia [11, 22] the cortex [23], and the hippocampus [24] or on the behavioral level [25, 26]. It can occur between two or three different modalities, for example auditory and visual [27, 28], visual and vestibular [29], auditory and tactile [30], or auditory, visual and somatosensory [11, 31, 32].

Having realized, though, that we did not have normative data about the modality-dependence of equivalence learning, we aimed to develop and introduce the auditory-guided and multisensory (audiovisual)-guided equivalence learning paradigms in order to compare the performance of healthy volunteers in the three (visual, auditory and multisensory) tasks. Special attention was paid to whether, during multisensory-guided learning, the earlier-described multisensory integration can be found on the behavioral level during multisensory-guided acquired equivalence learning. Earlier studies denoted that the multisensory information could facilitate learning. Multisensory information increases the learning speed in discrimination learning [33]. This occurs in selective learning tasks, too [34]. It is also known that the spatially coupled different modality stimuli could elicit more accurate orientation behavior than the spatially separated ones [35, 36]. We asked in the present study whether multisensory stimuli could similarly facilitate the acquired equivalence learning at a behavioral level. The general hypothesis of the present study was that multisensory guided associative learning is more effective in both its acquisition and test phases compared to those that employ unimodal visual and auditory guided paradigms.

## Methods

### Subjects

Altogether 151 healthy adult volunteers were involved in the research. All subjects were Caucasian. Only persons free of any ophthalmological, otological, neurological and psychiatric conditions were eligible. Intactness of color vision was tested by Ishihara plates prior to testing to exclude color blindness [37]. The potential subjects were informed about the background and goals of the study, as well as about the procedures involved. It was also emphasized that, given the lack of compensation or any direct benefit, the participants were free to quit at any time without any consequence (no one did so). Each participant signed an informed consent form.

The protocol of the study conformed to the tenets of the Declaration of Helsinki in all respects, and it was approved on several occasions by the Regional Research Ethics Committee for Medical Research at the University of Szeged, Hungary (50/2015-SZTE).

### The sensory guided associative learning paradigms

The tests were run on laptop computers (Lenovo T430, Fujitsu Siemens Amilo Pro V3505, Samsung Electronics 300e4z/300e5z/300e7z, Lenovo Yoga Y500) and with Sennheiser HD 439 closed, over-ear headphones for auditory and multisensory testing. The testing sessions took place in a quiet room with the subjects sitting at a standard distance (114 cm) from the computer screen. The M and X keys of the laptop keyboards were labeled left and right, respectively. One subject was tested at a time, and no time limit was set, so the subject could pay involuntary, undivided attention to learning. No forced quick responses were expected. The original visual associative learning test [4] written for iOS was slightly modified, translated to Hungarian and rewritten in Assembly (for Windows) with the written permission of Prof Catherine E. Myers (Rutgers University, NJ, USA), the corresponding author of the above-mentioned paper [4]. Beside the visually guided test, we also introduced an auditory and a multisensory (audiovisual) guided learning test, implemented in Assembly (for Windows). During the tests the participants had to associate two kinds of information referred to as antecedents and consequents. The participants were asked to learn associations of antecedent and consequent stimuli through trial and error during the task, and indicate their choice by pressing either the LEFT or RIGHT button of the laptop keyboard. The left or right button corresponded to the picture on each side of the computer monitor. All three paradigms were tested in two main phases, the acquisition and the test phases. In the acquisition phase the participant had to form associations between definite stimuli (equivalence acquisition) and the computer gave feedback about the success of the acquisition. A green check mark appeared on the screen to indicate a correct answer, while an incorrect answer was indicated by a red X. New associations were introduced one by one during the acquisition phase. The test phase, where no further feedback was provided, can be divided into two parts. Here the participant had to recall the previously-learned associations (in the retrieval part) and had to build new, hitherto-unknown but predictable acquisitions (in the generalization part) based on the rules learned in the acquisition phase. In the test phase the unknown new associations were presented mixed among the previously-learned ones. The subjects had to achieve a certain number of consecutive correct answers after the presentation of each new association (4 after the presentation of the first association, and 4, 6, 8, 10, 12 with the introduction of each new association, respectively) to be allowed to proceed. This ensured that the participants proceeded to the test phase only when they had memorized all the associations shown in the acquisition phase. Thus there were not a constant number of trials in the acquisition phase; the number depended on the performance of the subjects. On the other hand, the test phase consistently contained 48 trials, 36 of them previously-learned associations (retrieval part) and 12 new, previously not presented but predictable associations (generalization part).

#### Visual paradigm

**Fig 1:**
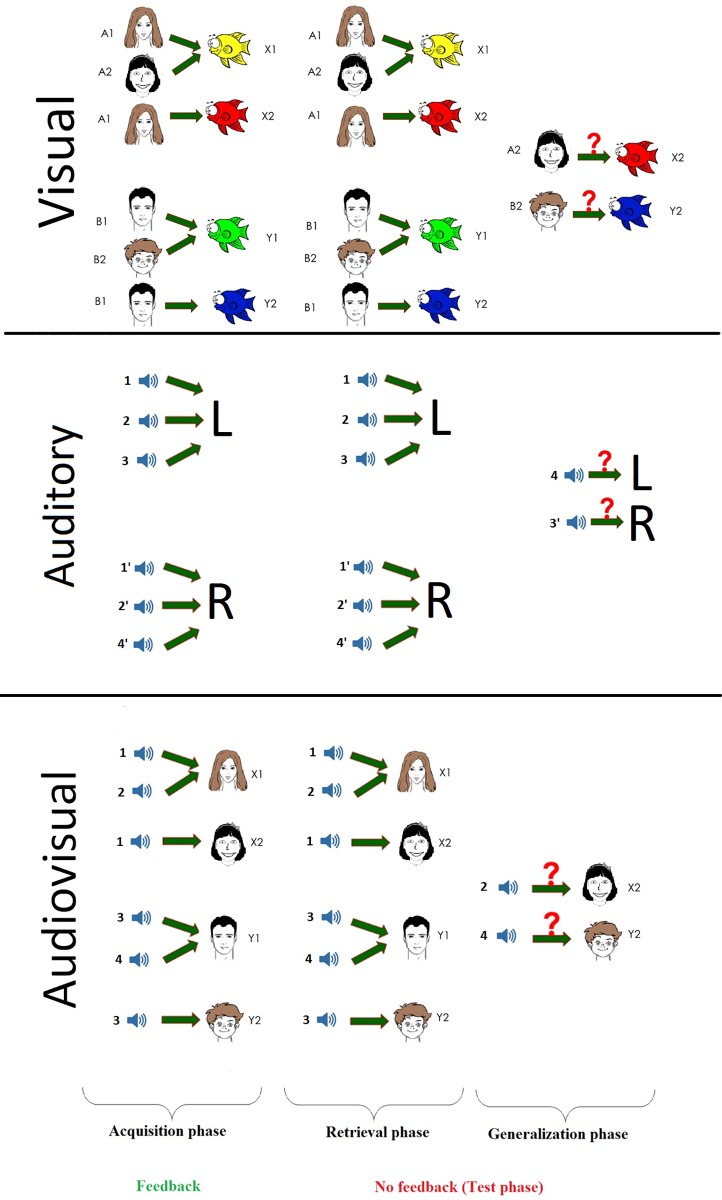
The schematic drawing of the applied visual, auditory and multisensory guided associative learning paradigms. See details in text.

Fig 1 illustrates the task in the three different paradigms.

The principle of the visual paradigm (Fig 1, top) is based on the Rutgers Acquired Equivalence Test (RAET) of Myers et al [4]. During each trial of the task the participants saw a face and a pair of fish, and were asked to choose which fish is matched with the given face. The faces were a girl, a boy, a man and a woman. The fish, which were of the same shape, had different colors: green, red, yellow and blue. There were four faces (A1, A2, B1, B2) and four fish (X1, X2, Y1, Y2) which could build eight pairs altogether. During the first two parts of the acquisition phase, the participants were expected to learn that when face A1 or A2 appeared, the correct answer was to choose fish X1 over fish Y1; given face B1 or B2, the correct answer was to choose fish Y1 over fish X1. This way the participants also learned that face A1 and A2 were equivalent in their consequent (face B1 and B2 likewise). In the next stage new consequents (X2, Y2) were introduced. Given face A1, participants had to choose fish X2 over Y2, and given face B1 they had to choose fish Y2 over X2. Until this point, participants had received feedback about the correctness of the decision. In the test phase, without any further feedback, the test presented the two new combinations beside the already-learned acquisitions. If the participants learned that A1 and A2 are equivalent, similarly to B1 and B2, they could generalize the previously-learned rule and could associate fish X2 with face A2 to (the fish that was associated with A1) and fish Y2 with face B2 to (the fish that was associated with face B1).

#### Auditory paradigm

In the auditory task the participants had to learn to associate sounds (antecedents) with the left or right buttons (L or R as consequents in Fig 1 middle), similarly to the visual paradigm, 8 pairs were built. Eight different sounds distributed into four pairs were used (in Fig 1 the following four sound pairs can be seen: sound 1 and sound 1’, sound 2 and sound 2’, sound 3 and sound 3’, sound 4 and sound 4’): two human voices of different genders (who said a word in Hungarian with neutral emotional tone), two animal sounds (a cat meowing, a dog barking), two sounds of musical instruments (a guitar, a piano), and two vehicle sounds (a motorcycle, an ignition key). The different sounds were randomly presented to each participant, so for example in one case, the sound 1 and sound 1’ mean the two animal sounds, in another case the sound 1 and sound 1’ mean the two vehicle sounds, etc. Each sound was 1.5 s long, and had the same intensity (SPL = 60 dB). The sound clips were played to the participants before the testing began through the headphones to each ear. The grouping was reflected in the distribution of sounds between the buttons: the first sound of a pair could be associated with to one key and the second sound of the same pair to the other key. The participants were expected to learn the pattern through trial and error, and apply it in the generalization phase of the task. During the acquisition phase the participants learned to associate two pairs of sound with buttons (altogether four associations), thus learning the pattern. Then the associations of one sound from each of the two remaining groups were learned. In the test phase, the participants had to generalize the correct association of the remaining two sounds. For feasibility reasons, which will be discussed in detail in the Discussion part of the paper, the auditory guided task does not totally correspond to the visual and multisensory guided ones. Although all of the learning tasks contain eight stimuli, in the auditory paradigm, in contrast to the visual and multisensory test where two visual or an auditory and a visual stimuli had to be associated, the sound has to be associated not with a second sound but with a particular button.

#### Multisensory paradigm

Apart from the stimuli, the experimental procedure of the multisensory (audiovisual) paradigm was exactly the same as the visual paradigm (Fig 1, bottom). Clearly-distinguishable sounds (one of the antecedents pairs used in the auditory paradigm: a cat’s meow, the sound of an ignition key, a note played by a guitar and a woman saying a Hungarian word with neutral emotional tone) served as antecedents (sound 1, sound 2, sound 3, sound 4) and faces were used as consequents (X1, X2, Y1, Y2). In each trial a sound (SPL = 60 dB) was played and two faces were presented to the participants, who had to learn which sound goes with which face. The stimuli were presented at the same time on the computer screen and through the headphones. The participants were asked to choose which face (left or right) is coupled with the given sound and were asked to press the corresponding button (left or right) on the keyboard. The auditory and visual components of the multisensory stimulus pairs were primarily semantically incongruent (except in the case of a woman’s voice being matched with a woman’s face).

### Data analysis

The trial numbers, the response accuracy (error ratios) and response times were analyzed in three groups in each paradigm: the acquisition phase, the retrieval part of the test phase and the generalization part of the test phase (minimal data set can be found in S1 File.). We registered the number of trials needed to complete the acquisition phase (NAT: Number of acquisition trials), the number of correct and incorrect choices during the acquisition phase, and the number of correct and incorrect answers for known and unknown associations during the retrieval and generalization parts of the test phase. Using these data, the error ratios were calculated: the ratio of the correct answers in the acquisition phase (ALER: Acquisition learning error ratio), in the retrieval part of the test phase (RER: Retrieval error ratio) and in the generalization part of the test phase (GER: Generalization error ratio). Reaction times (RT) in each phase for each answer were measured in ms with μs accuracy. The RTs were kept only within 3SDs of participants’ average.

To avoid a carry-over effect between paradigms, the different paradigms were recorded in a random order with each person.

The statistical analysis was performed in Statistica 13 (Dell Inc. USA) and G*Power 3.1.9.2. (Düsseldorf, Germany). One-way ANOVA was applied in order to compare the performances and the response times for each phase of the three learning paradigms. If the ANOVA analysis revealed significant difference among the values, the Tukey HSD post hoc test was applied to check the data pairwise. The effect sizes were calculated from means (in Statistica RMSSE, Root Mean Square Standardized Effect) because of the applied One-way ANOVA method. To determine the validity of the Miller’s race model [38, 39] an algorithm, developed earlier by Ulrich et al. [40] was applied on the visual, auditory and audiovisual response latencies in the generalization part of the paradigms.

## Results

Altogether 151 healthy volunteers participated in the study. Only a small minority of the participants (7/151) did not complete all three (visual, auditory, multisensory) paradigms. All of the participants could complete the visual paradigm, one of them could not learn the auditory, and six of them could not learn the multisensory associations. Only the performance and RT of those participants who completed all the three paradigms were further analyzed. After the further exclusion of the extreme outliers, 141 volunteers will be analyzed in detail (n_male_ = 41, age: 31.21±11.51 years, range: 18–72 years). The outliers were determined as a value above the mean +3SD (by the trial number in one of the paradigms).

### The performance in the three paradigms

**Fig 2:**
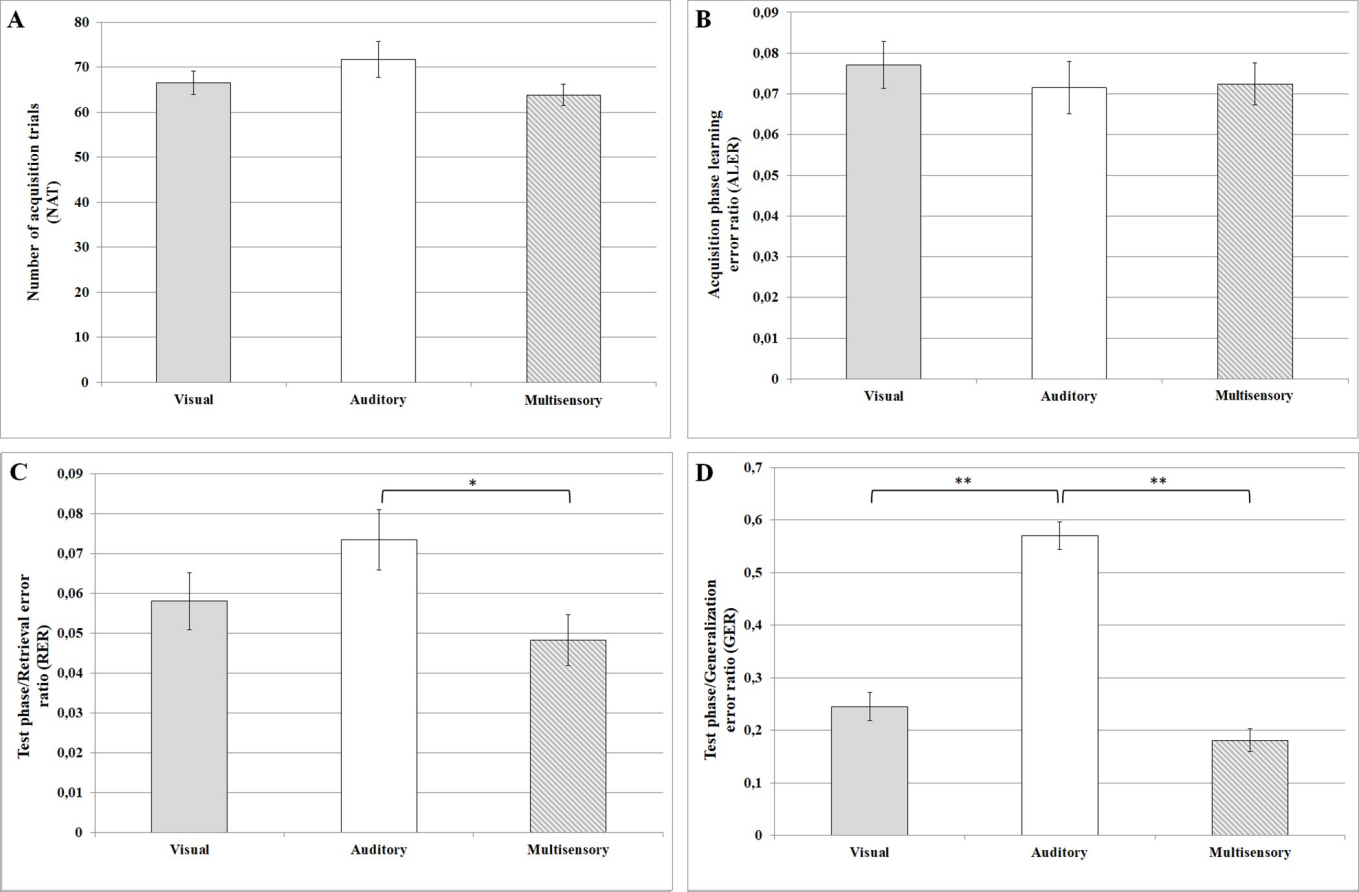
Performances in the sensory guided equivalence learning paradigms. (A) denotes the number of the necessary trials in the acquisition phase of the paradigm. (B) shows the error ratios in the acquisition phase of the paradigm. (C) and (D) denote the error ratios in the retrieval and generalization parts of the test phase, respectively. In each panel, the first column (light grey) shows the results in the visual paradigm, the second column (white) denotes the results in the auditory paradigm and the third column (grey-white striped) demonstrates the results in the multisensory (audiovisual) paradigm. Mean ± SEM values are presented in each column. The black stars denote the significant differences. The single star in part C represents a significant difference, where p<0.05; the two stars in part D represent strongly significant differences, where p<0.001.

The mean of the NAT necessary to learn the visual paradigm was 66.48 (range: 41–269, SEM: ±2.61, n = 141), in the case of the auditory paradigm it was 71.74 (range: 38–292, SEM: ±4.00, n = 141) and in the case of the multisensory paradigm it was 63.82 (range: 41–226, SEM: ±2.41, n = 141). The NATs did not differ significantly among the three (visual, auditory and multisensory) paradigms (ANOVA (F_(2, 420)_ = 1.7097, p = 0.18219) (Fig 2A).

In the visual paradigm the mean of the ALER was 0.0771 (range: 0–0.3333, SEM: ±0.0058), in the auditory paradigm it was 0.0715 (range: 0–0.359, SEM: ±0.0064) and in the multisensory paradigm it was 0.0724 (range: 0–0.347, SEM: ±0.0051). Similarly to the NATs, the ALERs showed no significant variation among the visual, auditory and multisensory paradigms (ANOVA F_(2, 420)_ = 0.26517, p = 0.76721 (Fig 2B)).

In the retrieval part of the test phase the RER was the highest in the auditory paradigm (mean: 0.07348, range: 0–0.4167, SEM: ±0.0075), it was moderate (mean: 0.0581, range: 0–0.4167, SEM: ±0.0072) in the visual paradigm and it was the lowest in the multisensory paradigm (mean: 0.0483, range: 0–0.4167, SEM: ±0.0064). There was a significant difference among these values (ANOVA: F_(2, 420)_ = 3.2659, p = 0.03913, Effect size: 0.0104, Power: 0.0420). The Tukey HSD post hoc test revealed that the multisensory RER was significantly lower than the auditory one (p = 0.030191), but there were no significant differences between the other combinations (Fig 2C).

The same trend can be observed in the generalization part of the test phase among the GERs. The GERs were the highest in the auditory paradigm (mean: 0.5703, range: 0–1, SEM: ±0.0264), while in the visual and multisensory paradigms they were nearly half of the auditory GER (visual mean: 0.2447, range: 0–1, SEM: ±0.0268, multisensory mean: 0.1809, range: 0–1, SEM: ±0.0217). There was a significant difference among these values (ANOVA F_(2, 420)_ = 9.4153, p<0.0001, Effect size: 0.2089, Power: 0.2444). The Tukey post hoc analysis revealed that both the visual and multisensory GERs were significantly lower than the auditory ones (visual vs. auditory p<0.001; multisensory vs. auditory p<0.001 (Fig 2D)).

In order to exclude the effect of learning during the tests, we investigated the effect of the sequence of the paradigms on performance. Altogether six different orders of paradigms were used, as their order was selected at random (Visual (V), Auditory (A), Multisensory (M), VMA, AVM, AMV, MVA, MAV). The statistical analysis (ANOVA) revealed no significant differences among the NATs, ALERs, RERs and GERs in the six possible orders.

### Latency of the correct trials in the three paradigms

**Fig 3:**
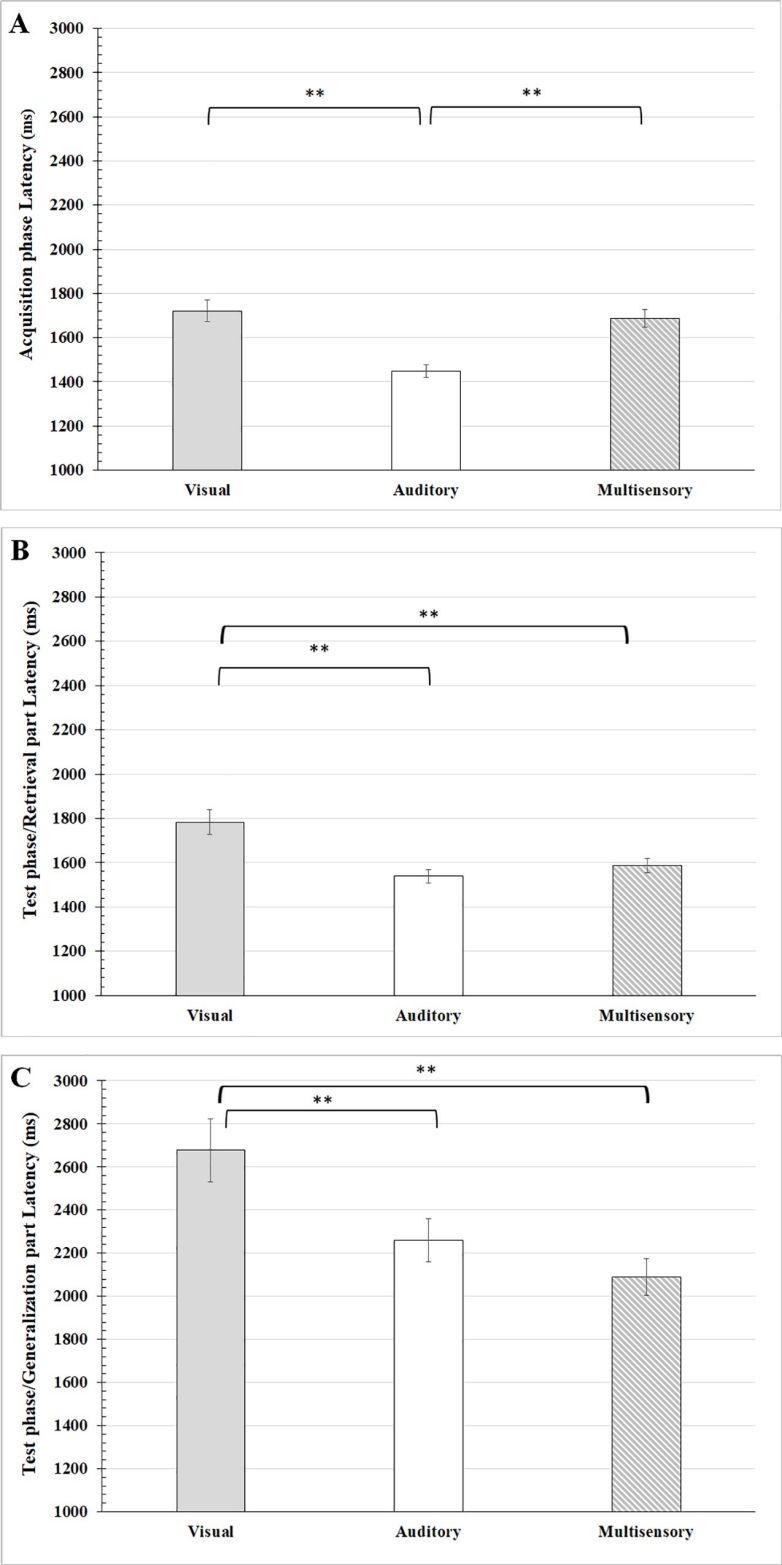
Response latencies in the sensory guided equivalence learning paradigms. (A) shows the response latencies in the acquisition phase of the paradigm, while (B) and (C) denote the response latencies in the retrieval and the generalization parts of the test phase, respectively. The ordinates show the latencies in millisecond (ms). Other conventions are the same as in Fig 2.

Fig 3 denotes the mean latencies of the correct trials in the acquisition phase and in the retrieval and generalization parts of the test phase in the three paradigms.

We compared the latency of the correct trials among the same phases of the different paradigms. The mean latency of the auditory correct trials in the acquisition phase was significant shorter (mean: 1447.86 ms, range: 850.43–3208.45 ms, SEM: ±28.92 ms, n = 141), than that of the visual (mean: 1721.21 ms, range: 841.63–3885.76 ms, SEM: ±49.31 ms, n = 141) and multisensory correct trials (mean: 1686.22 ms, range: 894.23–4017.16 ms, SEM: ±40.03 ms, n = 141; ANOVA (F_(2, 420)_ = 13.630, p<0.001, Effect size: 0.1218, Power: 0.9586, Tukey HSD post hoc between visual vs. auditory p<0.001, multisensory vs. auditory p<0.001) (Fig 3A).

Similarly to the acquisition phase, the mean latencies of the correct trials were different in the retrieval part of the test phase (ANOVA F_(2, 420)_ = 9.7615, p<0.001, Effect size: 0.105, Power: 0.9522, Tukey HSD post hoc visual vs. auditory p<0.001, visual vs. multisensory p = 0.0022). The mean latency of the visual correct trials was significantly longer (mean: 1782.65 ms, range: 825.81–4656.29 ms, SEM: ±55.39 ms, n = 141), than that of the auditory (mean: 1538.68 ms, range: 814.86–2884.62 ms, SEM: ±31.67 ms, n = 141) and multisensory correct trials (mean: 1585.58 ms, range: 893.58–2988.21 ms, SEM: ±32.86 ms, n = 141) (Fig 3B).

The mean latencies of the correct trials in the generalization part of the test phase differed significantly by modality (F_(2, 380)_ = 7.3734, p = 0.00072, Effect size: 0.2527, Power: 0.9503, Tukey HSD post hoc visual vs. auditory p = 0.0306, visual vs. multisensory p = 0.00053). In the generalization part of the test phase the mean latency of the visual correct trials was the longest (mean: 2677.81 ms, range: 940.8–10883.36 ms, SEM: ±145.95 ms, n = 133) and differed significantly from the other two (auditory mean: 2260.82 ms, range: 912.5–7633.5 ms, SEM: ±99.19 ms, n = 113; multisensory mean: 2089.71 ms, range: 882.58–6969.5 ms, SEM: ±84.12 ms, n = 137) (Fig 3C). While the mean multisensory response latency was the shortest in the generalization part of the test phase, the question arises whether this is because of the race between the visual and auditory modalities or because of the multisensory integration. In order to check this issue the race model inequality was analyzed (see S3 Fig). Based on these results the race model inequality can be held, which contradicts the effect of crossmodal multisensory integration on the audiovisual (multisensory) response latencies.

ANOVA analysis and the connected Tukey HSD post hoc analysis revealed that in all visual, auditory and multisensory paradigms the mean latency of the correct trials was significantly longer in the generalization part of the test phase than those in the acquisition phase or the retrieval part of the test phase. (The results of the detailed statistical analysis can be found here: visual paradigm F_(2, 412)_ = 33.19, p<0.000001, Effect size: 0.4326, Power: 0.9532, post hoc acquisition vs. generalization p = 0.00002, retrieval vs. generalization p = 0.00002; auditory paradigm F_(2, 392)_ = 58.63, p<0.000001 Effect size: 0.349, Power: 0.9532, post hoc acquisition vs. generalization p = 0.00002, retrieval vs. generalization p = 0.00002; multisensory paradigm F_(2, 416)_ = 22.176, p<0.000001, Effect size: 0.2167, Power: 0.9507, post hoc, acquisition vs. generalization p = 0.00002, retrieval vs. generalization p = 0.00002.)

## Discussion

The Rutgers Acquired Equivalence Test [4] was originally developed in order to learn about the visually guided associative learning of neurological patients with basal ganglia and hippocampus dysfunction. The test was applied later in cases of psychiatric disorders [41] and also to healthy subjects [6, 42]. Although both the basal ganglia and the hippocampi process not only visual but also multisensory information [10–13] the multisensory guided acquired equivalence learning had not been investigated before. As we recognized this absence we developed a multisensory (audiovisual) version of the associative learning test and were the first to investigate the basal ganglia and hippocampus mediated multisensory guided associative learning in healthy humans. We have to mention here that the aim of the study was not to measure directly the contribution of the involved structures to the paradigms. Thus, we could draw only indirect conclusions about the contribution of the basal ganglia and the hippocampi to the learning paradigms based on our psychophysical results and the results of previous publications in this field [4, 5, 7, 8]. This is a clear psychophysical study, which investigates the performance and the RT of healthy volunteers in different sensory guided associative learning paradigms.

The applied test can be divided into two parts irrespective of its modality. The first is the acquisition phase in which the subjects have to learn particular visual, auditory and multisensory stimulus combinations based on the feedback of the computer program. This process involves basal ganglia and the hippocampus. The association of new stimuli is dominated by the function of the basal ganglia [43, 44] and the coding and recall of associations are mainly a function of the medial temporal lobe [45]. Our results showed no significant difference between the performances (error ratio) in the unimodal visual, unimodal auditory and the combined audiovisual paradigms in the acquisition phase. Thus the modality of the stimuli does not affect the performance in this phase of the behavioral test. It is difficult to offer an explanation for this because it was described in several earlier studies that multisensory information could have more meaning than the sum of the unimodal ones [11, 46]. Multisensory integration has an important role not only in motor but also in cognitive functions of the brain. This multisensory facilitation plays a role in visual perception [47] object recognition [48, 49] emotional change recognition [50], face and voice recognition [51], or person recognition [52]. It affects the reaction time and accuracy of answers and the perceived threshold as well [27, 39, 53]. However, our results demonstrated absolutely no priority for the multisensory information in the acquisition phase of the applied associative learning paradigms. An explanation for this can be that such feedback based pair learning is a very old, conserved, and obligatory function which is so simple that the different modalities contribute to the association learning equally, and thus the multisensory information has no priority in these learning processes. This is in line with earlier findings that the basal ganglia, which are predominant in the acquisition phase of the associative learning test, are more active at the appearance of rare stimulus associations, which is not affected by modality [54]. It cannot be excluded that the semantic meaning of the stimuli could influence the performance in the learning paradigms. In a recent study it was demonstrated that semantically congruent audiovisual multisensory stimuli support multisensory integration [55]. In our experiment there was no attention paid to semantic contents because the task was the building of associations between the stimuli irrespectively of their meanings. As our stimuli were mainly semantically incongruent, this is another possible explanation for the lack of multisensory integration in the acquisition phase. At the behavioral level (opposed to the cellular level, [11] the presence of the multisensory integration is dependent on the level of attention and is not an automatic process [56].

The second part of the behavioral learning paradigm is the test phase, where the acquisitions learned earlier (retrieval) and hitherto not seen or heard pairs that were predictable by a previously deduced rule (generalization) were presented. The retrieval part of the test phase is dominated by the hippocampus-MT lobe system [45], and the generalization part of the test phase by the hippocampus and the basal ganglia [57]. Our results demonstrated that the performance was the most accurate (with the least incorrect answers) in the whole test phase of the multisensory guided paradigm although the multisensory performance differed significantly only from the auditory one, not the visual one. Thus, the multisensory-guided equivalence learning could be attributed mostly to visual learning, with the smaller benefit from the auditory modality. In the retrieval part, there was no difference between the unimodal tasks, but the performance in the multisensory task was significantly better than in the auditory one. Furthermore, in the generalization part, the performance in the unimodal visual task was significantly better than in the unimodal auditory one. Similarly, the performance in the multisensory task was significantly better than in the unimodal auditory one. We have to mention here the weakness of our study. The auditory guided task does not totally correspond to the visual and multisensory guided ones. Although all of the learning tasks contain eight stimuli, in the auditory paradigm the sound has to be associated not to a second sound but to a particular button on the keyboard, in contrast to the visual and multisensory tests where two visual stimuli or an auditory and a visual stimulus had to be associated. In an earlier draft of the auditory paradigm, we tried to apply one sound to each ear, but the participants would quickly become nervous and were not able to learn the acquisitions at all. However, the influence of this difference on the results cannot be explained by the auditory association to a keyboard button, as this seems to be an easier task than the visual and audiovisual associations. Nevertheless, the performances were worst in the auditory test.

The auditory and multisensory response latencies were not different but they were significantly shorter than the visual ones in the retrieval and generalization parts of the test phase. The most significant difference among the response latencies was in the generalization part of the test phase. If we compare the different phases of the paradigm, we can conclude that the generalization part of the test phase required the longest reaction times irrespective of the stimulus modality. This long decision time also supports that this is the hardest part of the applied cognitive learning task. We could not conclude that multisensory processing influences decision times, as would be suggested by Miller’s race model [39], which reported that a multisensory stimulus can elicit a faster response even without integration actually occurring. In contrast to this finding, in the acquisition and the retrieval part of the test phase the multisensory response did not have the shortest latency. On the other hand, in the generalization part of the test phase, the multisensory response latencies were the shortest. However, based on the visual, auditory and audiovisual response latencies the Miller’s race model was not violated [40]. This suggests that the shortest audiovisual response latency can be most probably explained by the race between the visual and auditory modalities and not by the multisensory (audiovisual) integration.

In summary, we can conclude that visual, auditory and multisensory guided association learning are similarly effective in healthy humans, which suggests that the primarily basal ganglia mediated acquisition phase is modality independent. On the other hand, in the test phase of the learning paradigm, which is dominated by the hippocampi, where the earlier-learnt acquisitions and hitherto not seen or heard but predictable associations are presented, the multisensory (audiovisual) stimuli elicited the best performance in the applied cognitive learning task. The test phase, especially its generalization part, seems to be a more difficult cognitive task than the acquisition phase, as the multisensory information processing could significantly improve the performance of the participants.

## Supporting information

S1 Fig Performances in the sensory guided equivalence learning paradigms. (A) denotes the number of the necessary trials in the acquisition phase of the paradigm. (B) shows the error ratios in the acquisition phase of the paradigm. (C) and (D) denote the error ratios in the retrieval and generalization parts of the test phase, respectively. In each panel, the first column (light grey) shows the results in the visual paradigm, the second column (white) denotes the results in the auditory paradigm and the third column (grey-white striped) demonstrates the results in the multisensory (audiovisual) paradigm. Mean ± SEM values are presented in each column. The black stars denote the significant differences. The single star in part C represents a significant difference, where p<0.05; the two stars in part D represent strongly significant differences, where p<0.001.
(DOCX)

S2 Fig Response latencies in the sensory guided equivalence learning paradigms. (A) shows the response latencies in the acquisition phase of the paradigm, while (B) and (C) denote the response latencies in the retrieval and the generalization parts of the test phase, respectively. The ordinates show the latencies in millisecond (ms). Other conventions are the same as in Suppl. 1.
(DOCX)

S3 Fig Test of the race model inequality. The figure represents the probability of cumulative frequency of response latencies in all three modalities (visual, auditory and audiovisual; x, y and z, respectively) and the sum of the two single modalities (x+y) in the generalization part of the test phase. The ordinate shows the latencies in milliseconds (ms) x 10^4^. Based on these results the race model inequality can be kept, which contradicts the effect of crossmodal multisensory integration on the audiovisual (multisensory) response latencies in the applied learning paradigm.
(DOCX)

S1 File Minimal data set. Worksheet titled “Results” contains the number of trials in the acquisition phase (NAT) and the number of errors in different phases of the tasks. Worksheet titled “RTs” shows the reaction times of all and the correct answers in different phases of visual, auditory and audiovisual paradigms.
(XLSX)
