# Supplementary material for: Multisensory guided associative learning in healthy humans
Source: PLoS One. 2019 Mar 12;14(3):e0213094. doi: 10.1371/journal.pone.0213094 (PMC6413907; doi:10.1371/journal.pone.0213094)
Supplement: S1 Fig — (A) denotes the number of the necessary trials in the acquisition phase of the paradigm. (B) shows the error ratios in the acquisition phase of the paradigm. (C) and (D) denote the error ratios in the retrieval and generalization parts of the test phase, respectively. In each panel, the first column (light grey) shows the results in the visual paradigm, the second column (white) denotes the results in the auditory paradigm and the third column (grey-white striped) demonstrates the results in the multisensory (audiovisual) paradigm. Mean ± SEM values are presented in each column. The black stars denote the significant differences. The single star in part C represents a significant difference, where p<0.05; the two stars in part D represent strongly significant differences, where p<0.001. (DOCX) [file pone.0213094.s001.docx]

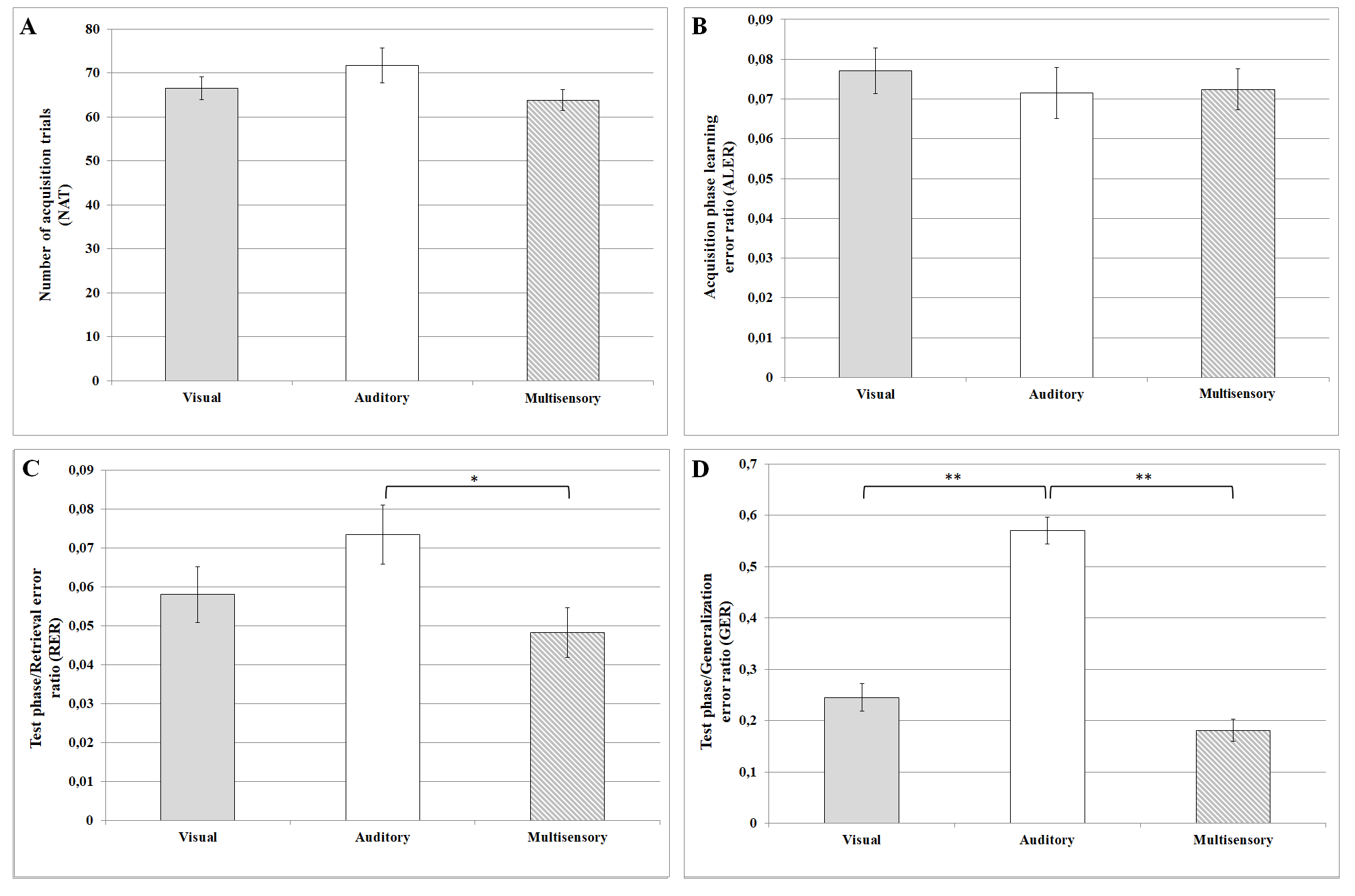


**Performances in the sensory guided equivalence learning paradigms.** (A) denotes the number of the necessary trials in the acquisition phase of the paradigm. (B) shows the error ratios in the acquisition phase of the paradigm. (C) and (D) denote the error ratios in the retrieval and generalization parts of the test phase, respectively. In each panel, the first column (light grey) shows the results in the visual paradigm, the second column (white) denotes the results in the auditory paradigm and the third column (grey-white striped) demonstrates the results in the multisensory (audiovisual) paradigm. Mean ± SEM values are presented in each column. The black stars denote the significant differences. The single star in part C represents a significant difference, where p<0.05; the two stars in part D represent strongly significant differences, where p<0.001.
