# Supplementary material for: Multisensory guided associative learning in healthy humans
Source: PLoS One. 2019 Mar 12;14(3):e0213094. doi: 10.1371/journal.pone.0213094 (PMC6413907; doi:10.1371/journal.pone.0213094)
Supplement: S2 Fig — (A) shows the response latencies in the acquisition phase of the paradigm, while (B) and (C) denote the response latencies in the retrieval and the generalization parts of the test phase, respectively. The ordinates show the latencies in millisecond (ms). Other conventions are the same as in Suppl. 1. (DOCX) [file pone.0213094.s002.docx]

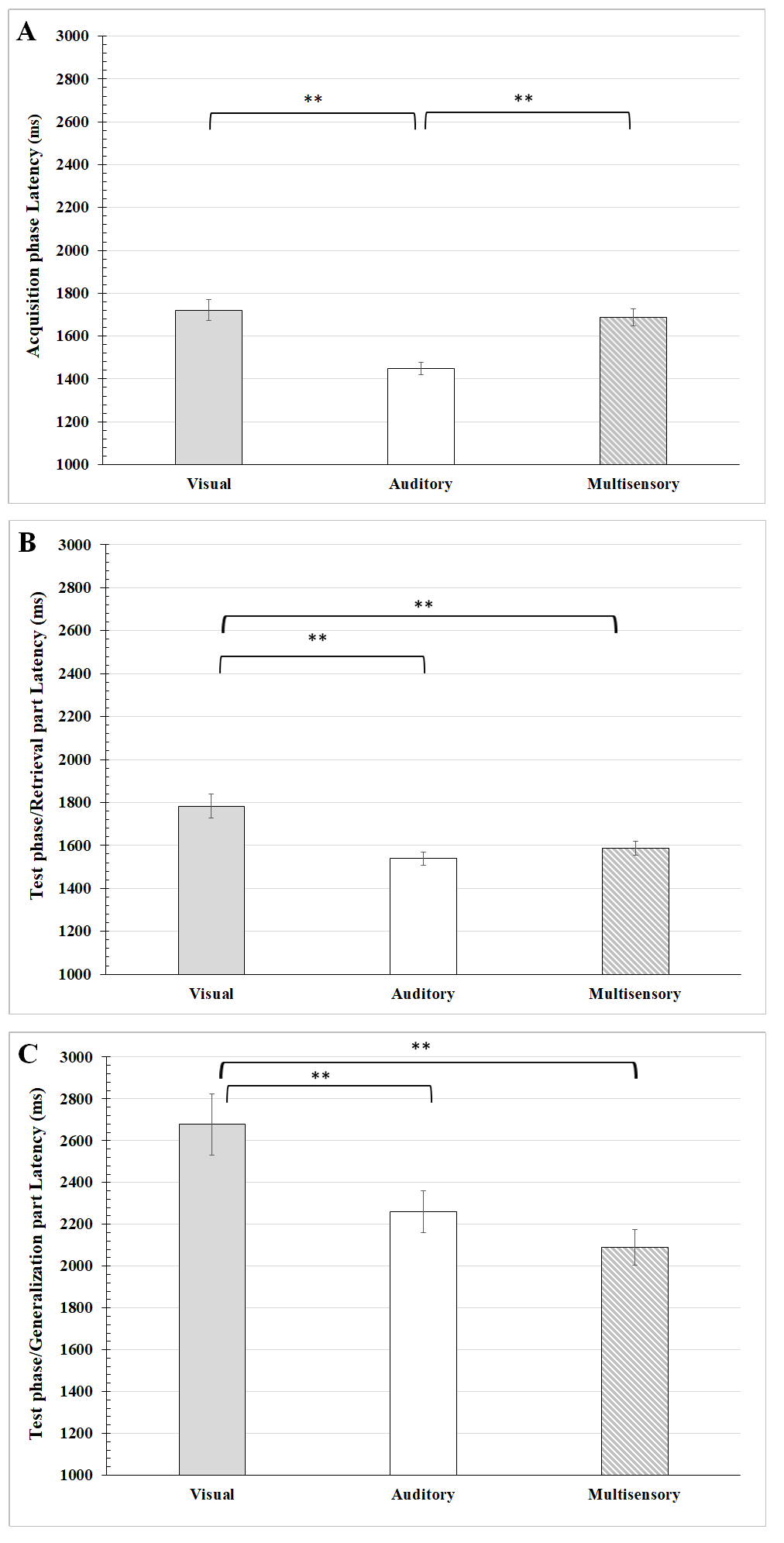


**Response latencies in the sensory guided equivalence learning paradigms**. (A) shows the response latencies in the acquisition phase of the paradigm, while (B) and (C) denote the response latencies in the retrieval and the generalization parts of the test phase, respectively. The ordinates show the latencies in millisecond (ms). Other conventions are the same as in Suppl. 1.
