# Supplementary material for: Multisensory guided associative learning in healthy humans
Source: PLoS One. 2019 Mar 12;14(3):e0213094. doi: 10.1371/journal.pone.0213094 (PMC6413907; doi:10.1371/journal.pone.0213094)
Supplement: S3 Fig — The figure represents the probability of cumulative frequency of response latencies in all three modalities (visual, auditory and audiovisual; x, y and z, respectively) and the sum of the two single modalities (x+y) in the generalization part of the test phase. The ordinate shows the latencies in milliseconds (ms) x 104. Based on these results the race model inequality can be kept, which contradicts the effect of crossmodal multisensory integration on the audiovisual (multisensory) response latencies in the applied learning paradigm. (DOCX) [file pone.0213094.s003.docx]

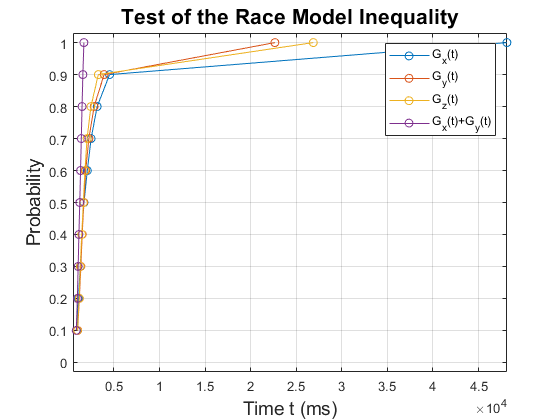


**Supplementary Figure 3 Test of the race model inequality.** The figure represents the probability of cumulative frequency of response latencies in all three modalities (visual, auditory and audiovisual; x, y and z, respectively) and the sum of the two single modalities (x+y) in the generalization part of the test phase. The ordinate shows the latencies in milliseconds (ms) x 10^4^. Based on these results the race model inequality can be held, which contradicts the effect of crossmodal multisensory integration on the audiovisual (multisensory) response latencies in the applied learning paradigm.
